# Association testing for binary trees—A Markov branching process approach

**DOI:** 10.1002/sim.9370

**Published:** 2022-03-09

**Authors:** Xiaowei Wu, Hongxiao Zhu

**Affiliations:** ^1^ Department of Statistics Virginia Tech Blacksburg Virginia USA

**Keywords:** association testing, binary tree, glioblastoma multiforme, Markov branching process

## Abstract

We propose a new approach to test associations between binary trees and covariates. In this approach, binary‐tree structured data are treated as sample paths of binary fission Markov branching processes (bMBP). We propose a generalized linear regression model and developed inference procedures for association testing, including variable selection and estimation of covariate effects. Simulation studies show that these procedures are able to accurately identify covariates that are associated with the binary tree structure by impacting the rate parameter of the bMBP. The problem of association testing on binary trees is motivated by modeling hierarchical clustering dendrograms of pixel intensities in biomedical images. By using semi‐synthetic data generated from a real brain‐tumor image, our simulation studies show that the bMBP model is able to capture the characteristics of dendrogram trees in brain‐tumor images. Our final analysis of the glioblastoma multiforme brain‐tumor data from The Cancer Imaging Archive identified multiple clinical and genetic variables that are potentially associated with brain‐tumor heterogeneity.

## 1 INTRODUCTION

Tree structured data are very common in nature, however the analysis of such data is challenging due to their non‐Euclidean topological structures. In recent years, many efforts have been devoted to develop statistical methods for modeling and analyzing tree structured data. Notable works include principal component analysis for trees,^1^, ^2^, ^3^, ^4^ Dyck path representation and analysis,^5^ and testing for dependence on tree structures.^6^ Despite the progress, there is still a pressing need for statistically sound and computationally efficient methods that are suitable for tree structured data arising from the real world.

One effective stochastic model for tree structures is the branching process. The branching process describes the size of an evolving population starting with a progenitor which splits into a random number of offspring according to *the offspring distribution*. Each of the offspring then produces its own offspring independently and such recurrent events (synchronized or asynchronized within each generation) form the entire population. Due to the self‐recurrence nature, branching processes are closely connected to trees and tree‐like graphs as the reproduction events indeed represent the birth of tree nodes. For this reason, branching processes have been widely used to study the characteristics of random trees, such as the percolation on trees^7^ and the height of various random search trees.^8^ Among the existing branching process models, the Galton‐Watson process—the very first stochastic model for population evolution, has been well studied and become the theoretical basis of other types of branching processes. However, the practical use of the Galton‐Watson process is often restricted by its discrete‐time assumption. As the continuous counterpart of the Galton‐Watson process, the continuous time Markov branching process (MBP) shares the same self‐recurrence (ie, branching) property with the Galton‐Watson process while allowing the lifetimes of the offspring to be independent, exponentially distributed random variables. Such a setting on offspring lifetimes makes the process Markovian, resulting in numerous applications in biological and physical sciences.

**FIGURE 1:**
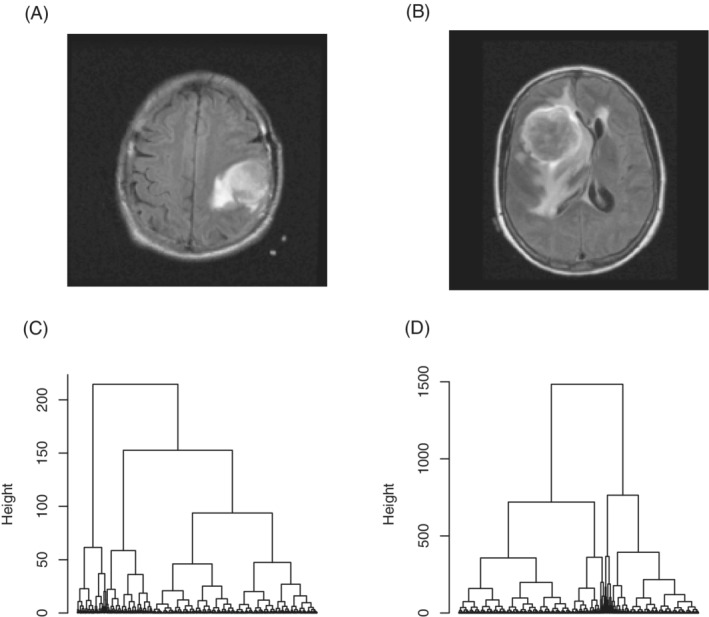
Binary trees generated from MRIs of two GBM patients in the TCIA database. (A, B) T2‐weighted MRI slices of the two patients, with bright areas indicating tumor regions; (C, D) the corresponding binary trees generated by performing hierarchical clustering on the pixel intensities of the segmented tumor images

In this article, we propose to model full binary trees (ie, trees in which every node other than the leaves has two children) with varying branch lengths by a binary fission MBP (bMBP). As a motivation example, Figure 1 demonstrates binary trees generated from two magnetic resonance images (MRIs) in The Cancer Imaging Archive (TCIA). In this figure, Panels A and B show brain‐tumor MRI slices of two patients with glioblastoma multiforme (GBM), and Panels C and D show the corresponding binary trees generated by performing hierarchical clustering on the pixel intensities of the segmented tumor images. The two example binary trees differ in their branch lengths, and we would like to explore factors, such as demographic and genetic variables, that may cause such differences. We believe that the binary tree structure carries important information about the characteristics of the brain tumors, which will be unveiled by the bMBP model. It is noteworthy that, due to the continuous branching lengths in the observed binary trees, the continuous‐time MBP should be a more appropriate model than the discrete‐time Galton‐Watson process. Our proposed approach for modeling binary trees facilitates convenient testing for association between binary trees, particularly size related characteristics of binary trees, and explanatory variables of interest. We develop procedures for association testing under this framework, for example, variable selection and estimation of covariate effects, and demonstrate the performance of these procedures by simulation studies and a real data application on brain‐tumor images of GBM. Our real data analysis identifies multiple covariates that are potentially associated with brain‐tumor heterogeneity.

The rest of this article is organized as follows. In Section 2, we first introduce the distribution of a special type of MBP—the birth and death process, from which we obtain the interarrival time distribution for the bMBP. We then present a generalized linear model (GLM) to associate binary trees with a set of covariates through the exponential rate parameter of the bMBP. In Section 3, we perform simulation studies to evaluate the performance of association testing methods under the GLM setting and check the applicability of the bMBP model by using semi‐synthetic brain‐tumor data. Section 4 describes detailed analysis of brain‐tumor image data in a real application, followed by conclusions and discussion in Section 5.

## 2 METHODS

**FIGURE 2:**
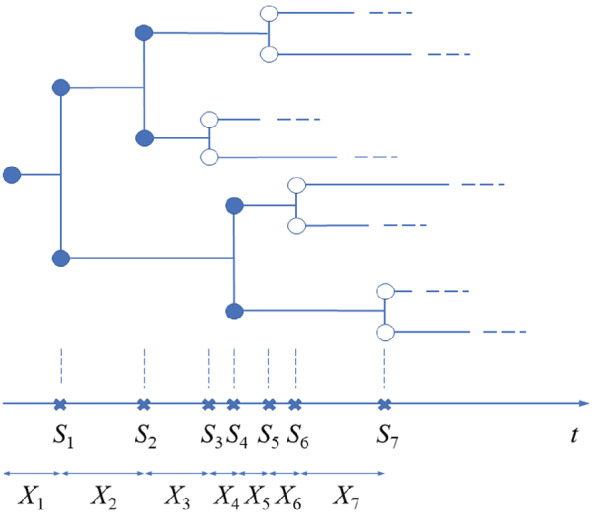
An illustration of the binary fission MBP and its interarrival times. Top: Binary tree as a sample path of the binary fission MBP; bottom: waiting times (denoted by $S_{i}$) and interarrival times (denoted by $X_{i}$) of the splitting events in the sample path

We consider full binary trees with varying branch lengths. This tree structure is commonly seen in practice, for example, in bifurcating phylogenetic trees in evolutionary biology, and more generally, in bifurcating trees generated from hierarchical clustering. Our study is motivated by analyzing the dendrogram tree obtained from hierarchical clustering the pixel intensities in tumor images (see Figure 1 for an example and Section 4 for detailed descriptions). Data with such a tree structure can be considered as realizations or sample paths of a bMBP, given that the Markov property is satisfied for the process. Since the reproduction pattern of the bMBP is fixed, that is, each particle produces exactly two children upon its death, the structure of the binary tree depends solely on the lifetime distribution of the MBP, making the inference easy and tractable. Figure 2 illustrates a sample path of the bMBP with initial population size one. It can be seen that, each particle lives, independently of others, for a random period of time and gives birth to two children at the end of its lifetime. Therefore, the bMBP is able to model a full binary tree whose branch lengths are determined by the lifetime distribution of the particles, or more precisely, are sampled independently from exponential distribution.

With this binary fission MBP interpretation, size related characteristics of the binary trees are determined by the MBP parameters, and these parameters can be inferred from the observed summary statistics of the binary trees. For example, we may use the observed waiting or interarrival times of the splitting events (in other words, death events of the parent particles or birth events of the children particles) to estimate the exponential rate of the lifetime, thereby shedding light on the shape of the binary tree in a probabilistic way. For better understanding, we show in the bottom of Figure 2 the waiting times $S_{i}$ and interarrival times $X_{i}$ for the bMBP sample path. In the following two sections, we present the distribution of the interarrival times in a bMBP, and from which we develop procedures for testing associations between binary trees and covariates.

### 2.1 Distribution of interarrival times in a binary fission MBP

The continuous time MBP is specified by two parameters, the offspring distribution and the exponential rate of the lifetime distribution.^9^ As an example, let Y(t) denote a birth and death process—a special continuous time MBP whose offspring distribution has a probability generating function (PGF) $f(s)=p+(1-p)s^{2}$, that is, at the end of its lifetime, each particle will die with probability *p*, and will give birth to two offspring with probability 1−p. Denoting the exponential rate by λ, by solving the Kolmogorov backward equation $\frac{\partial }{\partial t}F(s,t)=\lambda [F{\left(s,t\right)}^{2}-F(s,t)]$ where F(s,t) is the PGF of Y(t), the probability mass function (PMF) of Y(t), assuming unit initial population size, can be obtained explicitly^10^, ^11^

(1) $$\begin{array}{ll} P(Y(t)=n)=\alpha q{\left(1-q\right)}^{n-1},\;n=1,2,\ldots ,\;\forall t>0, & \end{array}$$

where $q=(1-2p)e^{-ct}/\left[1-p-pe^{-ct}\right]$, c=λ(1−2p) denotes the Malthusian parameter, and $\alpha =qe^{ct}$. In other words, Y(t) follows a generalized geometric distribution.^12^, ^13^

Let us further consider the special case of binary fission MBP in which case p=0. The PMF expression in (1) simplifies to a geometric distribution with parameter $e^{-\lambda t}$, that is, $Y(t)∼\text{geo}(e^{-\lambda t}),\forall t>0$. Therefore, the cumulative distribution function (CDF) of Y(t) is

(2) $$\begin{array}{ll} P(Y(t)\leq n)=1-{\left(1-e^{-\lambda t}\right)}^{n},\;n=1,2,\ldots ,\;\forall t>0. & \end{array}$$

From (2), the waiting and interarrival time distributions of the bMBP can be derived. Define a counting process {N(t),t≥0} so that the events in N(t) correspond to the splitting events in the bMBP. It is easy to see that N(t)=Y(t)−1 and N(0)=0. Let $S_{n}$ be the waiting time until the *n*th event occurs in the N(t) process (see illustration in the bottom of Figure 2). Since $\left\{S_{n}\leq t\}\Leftrightarrow \{N(t)\geq n\right\}$, the CDF of $S_{n}$ can be obtained

$$\begin{array}{ll} P(S_{n}\leq t)=P(N(t)\geq n)=1-P(Y(t)\leq n)={\left(1-e^{-\lambda t}\right)}^{n},\;n=1,2,\ldots ,\;\forall t>0. & \end{array}$$

Further, for n>1, let $X_{n}$ denote the time between the (n−1)st and the *n*th events in the N(t) process, that is, $X_{n}=S_{n}-S_{n-1}$, and let $X_{1}=S_{1}$. Such a sequence $\left\{X_{n},n\geq 1\right\}$ is the sequence of interarrival times in the N(t) process (see illustration in the bottom of Figure 2). The proposition below shows that the interarrival times $X_{n}$ follow independent exponential distributions.

Proposition 1 *The interarrival times* $X_{n}$ *of a binary fission MBP are independent of each other and* $X_{n}∼\exp(n\lambda ),n\geq 1$.

For a binary fission MBP, we have $P(S_{n}\leq t)={\left(1-e^{-\lambda t}\right)}^{n}$. Denote the moment generating function (MGF) of $S_{n}$ by $M_{S_{n}}(\nu )$, then

MSn(ν)=∫0∞eνt·nλe−λt(1−e−λt)n−1dt=nλ∫0∞e(ν−λ)t∑k=0n−1(−1)kn−1ke−kλtdt=nλ∫0∞∑k=0n−1(−1)kn−1ke[ν−(k+1)λ]tdt=nλ∑k=0n−1n−1k(−1)k(k+1)λ−ν=∏k=1nkλkλ−ν.

Since $M_{S_{n}}(\nu )$ factorizes to the product of *n* exponential MGF's, each with rate parameter kλ, we conclude that $X_{n}$'s are independent of each other and $X_{n}∼\exp(n\lambda ),n\geq 1$.

We note that, the above result of interarrival time distribution can also be seen from the fact that, in the bMBP, $X_{n}$ is the smallest one among *n* i.i.d. exponential lifetimes starting at $S_{n-1}$, with the memoryless property taken into account.

Based on Proposition 1, statistical inference about the unknown parameter λ of the bMBP can be done by using the observed interarrival times. It should be noted that, given the sample path of the bMBP, the interarrival times play an equivalent role to the lifetimes—they both are sufficient statistics for the inference of λ, however, the former is more useful in practice as the splitting events can always be observed but the lineage of the tree nodes may not be known. More details about inferring λ from the observed interarrival times and using a simulation study to evaluate the inference results can be found in Appendices A and B, respectively.

### 2.2 Modeling associations between binary trees and covariates

In order to investigate how explanatory variables influence binary trees, particularly size related characteristics of binary trees such as the frequency of splitting, we treat binary trees as realizations of independent bMBPs and link the lifetime parameter λ with the covariates of interest.

Let $Y_{i}(t)$ be the *i*th bMBP whose growth is determined by the exponential rate parameter $\lambda_{i}$, i=1,…,m. We propose the following GLM framework

(3) $$\begin{array}{ll} Y_{i}(t) & \;\overset{\text{ind}}{∼}\;\text{MBP}(\lambda_{i}),\;1\leq i\leq m, \end{array}$$

(4) $$\begin{array}{ll} \lambda_{i} & =\beta_{0}+\overset{q}{\underset{k=1}{\sum }}\beta_{k}Z_{ik}, \end{array}$$

where $Z_{ik}$ denotes the *k*th covariate associated with $Y_{i}(t)$, 1≤k≤q, and $\beta_{k}$'s are the corresponding coefficients. Let $X_{ij}$ denote the *j*th interarrival time of $Y_{i}(t)$. By Proposition 1, we may replace (3) by

(5) $$\begin{array}{ll} X_{ij}\;\overset{\text{ind}}{∼}\;\exp(j\lambda_{i}),\;1\leq j\leq n. & \end{array}$$

With this setup, we have treated the interarrival times of the bMBPs rather than the bMBPs themselves as the responses. As seen in Sections 3.2 and 4, in the real application of brain‐tumor image data, the response variable $X_{ij}$ is obtained by first performing hierarchical clustering to the pixel intensities of the segmented tumor region for the *i*th patient, calculating the waiting times for the corresponding dendrogram tree, and then extracting the *j*th interarrival time. $Z_{ik}$ refers to the *k*th covariate (demographic, trait‐related, or genetic variables) of the *i*th patient. Note that for any bMBP, theoretically the number of interarrival time goes to infinity. However, the number of practically observed splitting events in real data, *j*, is always finite. Therefore, we give it an upper bound *n*. In other words, despite the infinite many splitting events in the bMBP, we assume that only the first *n* will be considered in the actual sample paths. Association testing based on the above GLM model can be done in various ways. Here we incorporate two commonly used approaches: stepwise regression and Lasso for GLM.

#### 2.2.1 Stepwise regression (backward elimination)

Stepwise regression involves adding or removing potential explanatory variables in succession according to some predefined criterion. One form of stepwise regression is called background elimination, or sequential backward selection, which includes all available variables initially and then tests the deletion of each variable one by one. The process stops when the variable selection criterion, such as the likelihood ratio test (LRT) criterion or Akaike information criterion (AIC) is satisfied.

For our GLM framework (5) and (4), we may write the log‐likelihood as

(6) $$\begin{array}{ll} \ell (\vec{\beta }) & =\overset{m}{\underset{i=1}{\sum }}\overset{n}{\underset{j=1}{\sum }}\left[\ln\left(j\lambda_{i}\right)-j\lambda_{i}x_{ij}\right]\\ & =m\ln n!+n\overset{m}{\underset{i=1}{\sum }}\ln\left(\beta_{0}+\overset{q}{\underset{k=1}{\sum }}\beta_{k}Z_{ik}\right)-\overset{m}{\underset{i=1}{\sum }}\left[\left(\beta_{0}+\overset{q}{\underset{k=1}{\sum }}\beta_{k}Z_{ik}\right)\overset{n}{\underset{j=1}{\sum }}jx_{ij}\right]. \end{array}$$

Based on (6), the LRT or AIC criterion can be calculated in the backward elimination process. Appendix C provides a simulation study to evaluate stepwise regression by the LRT criterion in terms of type I error rate and empirical power.

Besides variable selection, estimating regression coefficients is another step in the backward elimination process. Let $\hat{\beta }={\left[{\hat{\beta }}_{0},{\hat{\beta }}_{1},\ldots ,{\hat{\beta }}_{q}\right]}^{T}$ denote the MLE of the unknown regression coefficients. By the invariance property of MLE,^14^

$$\begin{array}{ll} \hat{\beta }={\left(Z^{T}Z\right)}^{-1}Z^{T}\frac{n}{\sum_{j=1}^{n}jX_{j}}, & \end{array}$$

where $Z=[Z_{ij}]$ is an m×q matrix of the covariates (similar to the design matrix in a linear regression model) and $X_{j}={\left[X_{1j},X_{2j},\ldots ,X_{mj}\right]}^{T}$ is a vector containing the *j*th interarrival times of all *m* bMBPs. As a side note, the exact confidence intervals for the regression coefficients can be obtained (see Appendix A.1 for details).

#### 2.2.2 Lasso for GLM

Another popular approach for testing association in the GLM framework (5) and (4) is by Lasso for GLM. The objective of Lasso for GLM is to solve

$$\begin{array}{ll} \underset{\vec{\beta }}{\min}-\ell (\vec{\beta })\;\text{subject to}\;\overset{q}{\underset{k=1}{\sum }}|\beta_{k}|\leq t, & \end{array}$$

where *t* is a prespecified free parameter that determines the degree of regularization. Equivalently, such an objective function is the penalized negative log‐likelihood function, and in our context is

$$\begin{array}{ll} \underset{\vec{\beta }}{\min}-\ell (\vec{\beta })+\alpha ||\vec{\beta }|{|}_{1}, & \end{array}$$

where the tuning parameter α controls the strength of the penalty term in Lasso. Note that, our model (5) and (4) indeed specifies a GLM in the family of Gamma distribution with a reciprocal link function.

## 3 SIMULATION STUDIES

We design two simulation studies to assess the proposed model and inference procedures. Simulation 1 uses data generated from the bMBP model to calculate the accuracy of variable selection by backward elimination and Lasso for GLM. Simulation 2 uses semi‐synthetic data—data that resemble real brain‐tumor image—to check the applicability of the bMBP model.

### 3.1 Accuracy of variable selection by backward elimination and Lasso for GLM

In this simulation study, binary trees are generated as bMBP sample paths for 1000 patients with exponential rate parameter $\lambda_{i}=\beta_{0}+\sum_{k=1}^{q}\beta_{k}Z_{ik},1\leq i\leq 1000$. Here, the total number of covariates q=20 (similar to that in the real application, as seen from Section 4), among which a subset of 2,4,…,18 variables are associated with the observed binary trees. The covariates are all generated from folded normal distribution (ie, the distribution of the absolute value of a normal random variable) with mean μ=0 and standard deviation σ=0.1, and the coefficients $\beta_{k},0\leq k\leq q$ are sampled uniformly from [0.5, 1.5].

For the number of associated variables varying in {2,4,…,18}, we repeat the simulation 1000 times, and in each simulation we compare the set of selected variables with the set of associated variables to calculate the accuracy of variable selection. Different criteria may be used for comparing these two sets, for example, the Jaccard similarity coefficient (ie, the ratio of intersection over union) and the $F_{1}$ score (ie, the harmonic mean of precision and recall). Here, to demonstrate the “hit” and “false alarm” separately in variable selection, we use the average true positive rate (TPR) and average false positive rate (FPR). By treating the selected variables as “positive,” the TPR/FPR of a variable can be defined as the frequency of selecting an associated/unassociated variable in repeated simulations. Therefore, the average TPR describes how likely each of the associated variables is selected, and on the other hand, the average FPR describes how likely each of the unassociated variables is selected. Table 1 lists the accuracy of variable selection, together with the accuracy of prediction on the exponential rate parameter $\lambda_{i}$. For backward elimination, we use AIC as the stopping criterion, that is, the iterative process of narrowing down from the initial set of all variables will stop when no candidate model achieves an AIC smaller than the current model. When using Lasso for GLM, the optimal tuning parameter is determined by 10‐fold cross validation according to the “one‐standard‐error” rule.^15^, ^16^ The prediction accuracy is measured by the average of the coefficient of variation of the root‐mean‐square deviation, abbreviated by average CV(RMSD), over the 1000 trees, where the RMSD for each $\lambda_{i}$ in the total *T* simulations is defined by $\text{RMSD}=\sqrt{\sum_{j=1}^{T}{\left({\hat{\lambda }}_{i}-\lambda_{i}\right)}^{2}/T}$, and the coefficient of variation of the RMSD is $\text{RMSD}/{\bar{\lambda }}_{i}$.

**TABLE 1:** Evaluation of variable selection when the total number of variables is 20

	Backward elimination			Lasso for GLM		
# of asso vars (out of 20)	Ave TPR	Ave FPR	Ave CV (RMSD)	Ave TPR	Ave FPR	Ave CV (RMSD)
2	1	0.1056	0.0494	1	0.0006	0.0634
4	1	0.0996	0.0495	1	0.0043	0.0662
6	1	0.1082	0.0497	1	0.0138	0.0651
8	1	0.1020	0.0498	1	0.0273	0.0663
10	1	0.1098	0.0501	1	0.0537	0.0673
12	0.9987	0.1069	0.0503	0.9876	0.0599	0.0657
14	0.9999	0.1083	0.0506	0.9994	0.0845	0.0667
16	1	0.1080	0.0506	0.9998	0.1225	0.0671
18	0.9998	0.1030	0.0509	0.9993	0.1395	0.0676

In addition, we include the results for q=10 and q=100 in Tables 2 and 3, respectively, corresponding to the scenarios of small and large total number of variables. From these results, we see that, when the total number of variables is small (q=10 or 20), both backward elimination and Lasso for GLM achieve high average TPR (nearly 1); the average FPR of backward elimination appears to be stable around 0.1 but Lasso for GLM has lower average FPR at most of time which increases with the number of associated variables. When the total number of variables is large (q=100), the average TPR for both methods are still comparable, while starting to drop as the number of associated variables increases; the average FPR for backward elimination still fluctuates around 0.1 whereas for Lasso for GLM, the false alarm climbs quickly as the number of associated variables increases. These observations show that both backward elimination and Lasso for GLM are able to identify associated variables especially when the total number of variables is at small or moderate level. We also note that, Lasso for GLM tends to select more parsimonious models in comparison to backward elimination, which can be seen from their average CV(RMSD). After all, selecting more variables generally helps make more accurate predictions.

**TABLE 2:** Evaluation of variable selection when the total number of variables is 10

	Backward elimination			Lasso for GLM		
# of asso vars (out of 10)	Ave TPR	Ave FPR	Ave CV (RMSD)	Ave TPR	Ave FPR	Ave CV (RMSD)
1	1	0.1053	0.0489	1	0.0001	0.0631
2	1	0.0996	0.0489	1	0.0001	0.0639
3	1	0.1069	0.0491	1	0.0036	0.0652
4	1	0.1055	0.0493	1	0.0025	0.0673
5	1	0.0978	0.0490	1	0.0090	0.0653
6	1	0.0995	0.0493	1	0.0160	0.0658
7	1	0.0933	0.0495	1	0.0283	0.0657
8	1	0.1130	0.0496	1	0.0475	0.0671
9	1	0.1060	0.0496	1	0.0270	0.0663

**TABLE 3:** Evaluation of variable selection when the total number of variables is 100

	Backward elimination			Lasso for GLM		
# of asso vars (out of 100)	Ave TPR	Ave FPR	Ave CV (RMSD)	Ave TPR	Ave FPR	Ave CV (RMSD)
10	0.9611	0.1067	0.0542	0.9977	0.0216	0.0666
20	0.9929	0.1049	0.0543	0.9982	0.0788	0.0695
30	0.9743	0.1081	0.0556	0.9786	0.1124	0.0694
40	0.9312	0.1064	0.0563	0.9016	0.1710	0.0693
50	0.9473	0.1065	0.0570	0.9571	0.2600	0.0707
60	0.8748	0.1127	0.0582	0.8743	0.2552	0.0703
70	0.8174	0.1108	0.0594	0.8449	0.2858	0.0705
80	0.7876	0.1126	0.0603	0.8313	0.3046	0.0709
90	0.7097	0.1134	0.0611	0.7537	0.2903	0.0706

### 3.2 Simulating semi‐synthetic brain‐tumor image data to check the applicability of the bMBP model

**FIGURE 3:**
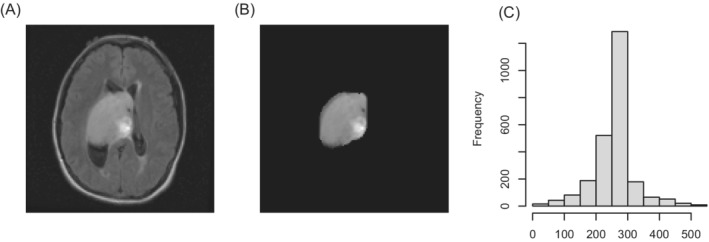
An example of the real brain‐tumor image data. (A) T2‐weighted MRI slice; (B) tumor image; (C) histogram of pixel intensities in tumor image

An example of the real brain‐tumor image data is given in Figure 3, where Panel A shows a single 2D slice from a T2‐weighted MRI of a patient diagnosed with GBM, Panel B is the segmented tumor image after applying a mask, and Panel C shows the histogram of the pixel intensities in the tumor image. We use the tumor image in Panel B as a template and generate pixel intensities according to scaled beta distributions; the scaling was done to make sure the range of the simulated pixel intensities matches the real data. Using the simulated pixel intensities, we perform hierarchical clustering, and treat the dendrograms as binary trees. We consider these binary trees as realizations of bMBPs and extract the interarrival times, based on which we further check whether the exponential lifetime assumption of the MBP model is satisfied.

**FIGURE 4:**
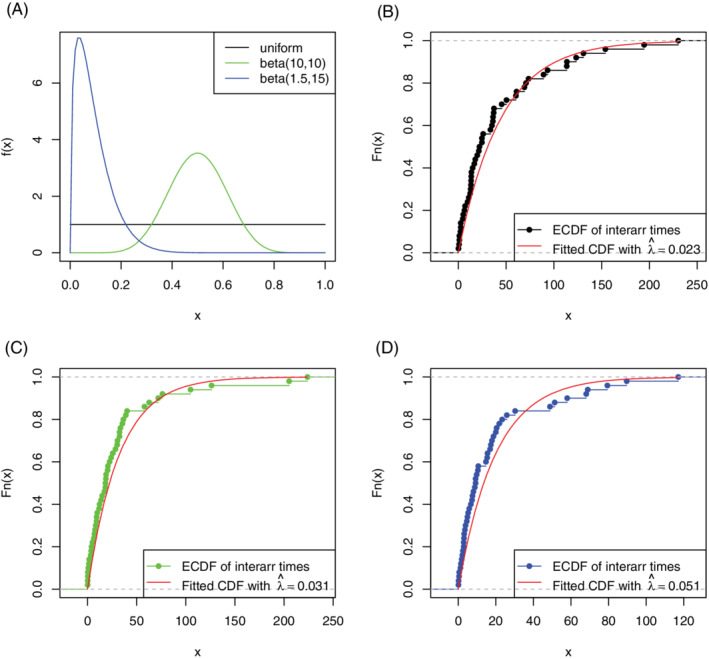
(A) Three pixel intensity distributions used in the semi‐synthetic brain‐tumor images; (B‐D) goodness‐of‐fit check by comparing empirical CDF of the scaled interarrival times $jX_{j}$ vs CDF of the fitted exponential distribution, when pixel intensities follow (B) uniform distribution, (C) beta(10, 10) distribution, and (D) beta(1.5, 15) distribution

Specifically, we let the pixel intensities follow three distributions: uniform, beta(10, 10), and beta(1.5, 15), whose probability density functions are plotted in Figure 4, Panel A. Each beta distribution results in one dendrogram and one set of interarrival times. We denote the *j*th interarrival time by $X_{j}$, and rescale $X_{j}$ by its index. That is, we calculate $jX_{j}$ for 1≤j≤n where *n* is the given upper bound for the number of splitting events. In this simulation, we set n=50. If such dendrogram trees can be modeled by the bMBP, by (5), the scaled interarrival times should be identically distributed as exponential. We then plot the empirical CDFs of the scaled interarrival times in contrast with the CDFs of the fitted exponential distributions (with rate parameter λ estimated from $X_{j}$'s). The CDF plots corresponding to the three pixel intensity distributions are shown in Figure 4, Panels B to D, respectively.

The plots in Figure 4 demonstrate that the interarrival times can be well fitted with exponential distributions. (The Kolmogorov‐Smirnov test *P*‐values corresponding to uniform, beta(10, 10), and beta(1.5, 15) pixel intensity distributions are 0.4766, 0.3846, and 0.1406, respectively.) Therefore, the bMBP model is applicable to the binary trees generated in this simulation study. This provides us the rationale for implementing our modeling and inference framework in the real data analysis in Section 4. Moreover, this simulation study also provides two additional remarks:

1. In tumor images, since pixels with similar intensities are likely to be originated from the same etiological source, the shape of the pixel intensity distribution, in particular, the *spikiness*, carries information about tumor heterogeneity. Therefore, the hierarchical clustering dendrogram on pixel intensities may reveal the latent ordering of cells developing tumor. In other words, information about tumor heterogeneity is indicated in the dendrogram tree, which, when modeled by the bMBP model, can be characterized by the exponential rate parameter. In general, spiky distribution usually exhibits lower variability of pixel intensities, thus corresponds to lower tumor heterogeneity.
2. As the pixel intensity distribution changes from uniform to more spiky beta distributions, the fitted exponential rate parameter increases. We note that, the spikiness of these three distributions can be measured by their differential entropies.^17^ From uniform, beta(10, 10), to beta(1.5, 15), we observe deficiency in differential entropy: 0, −0.798, to −1.462, implying a decrease in the level of “surprise” or “uncertainty” inherent in the intensity of a randomly chosen pixel in the tumor image. The intuitive explanation seems fairly simple—spiky distribution of pixel intensities encourages clear‐cut clusters that are easy to distinguish at earlier time, thereby yielding on average shorter interarrival times in the bMBP model. Therefore, we may further conclude that larger exponential rate parameter indicates lower tumor heterogeneity.

In practice, the pixel intensity distribution of the tumor image may be more complicated than unimodal beta distributions. Therefore, we need to always check the goodness‐of‐fit of the scaled interarrival times to the fitted exponential distribution to guarantee the applicability of the bMBP model. Once the bMBP model is applicable, more simulations and supervised learning methods are desirable to validate the relation between the spikiness of the pixel intensity distribution and tumor heterogeneity.

## 4 APPLICATION TO REAL BRAIN‐TUMOR IMAGE DATA

Tumor heterogeneity represents the distinct morphological and phenotypic patterns exhibited in tumor cells. In brain‐tumor image data, the heterogeneity of brain‐tumor is often seen from the similarity in pixel intensities. We are particularly interested in characterizing the link between brain‐tumor heterogeneity and clinical/genetic variables. This allows us to better understand the causes and progression of brain‐tumors. For this purpose, we apply the proposed bMBP model to real brain‐tumor image data to select variables that are associated with the heterogeneity of brain‐tumor and estimate their effects.

The brain‐tumor image data contain presurgical, T1‐weighted post‐contrast, and T2‐weighted fluid attenuated inversion recovery (FLAIR) MRIs of 63 patients (21 female and 42 male) diagnosed with GBM, an aggressive brain cancer. The raw image data are publicly available on TCIA (https://www.cancerimagingarchive.net). A total of 19 covariates can be downloaded from cBioPortal (http://www.cbioportal.org), including the patients' demographic variables (age, gender), trait‐related variables (Karnofsky score, months of disease‐specific survival, overall survival status, FLAIR volume, classical, mesenchymal, neural, proneural), and several genetic markers (*EGFRmut*, *IDH1mut*, *DDIT3*, *EGFR*, *KIT*, *MDM4*, *PDGFRA*, *PIK3CA*, *PTEN*) which have been considered important GBM driver genes.^18^ The raw images were preprocessed to extract three‐dimensional (3D) tumor volumes. Details of the preprocessing procedure, including spatial registration, bias correction, and tumor‐region segmentation, can be found in literature.^19^

In this study, we use T2‐weighted images and attempt to characterize tumor heterogeneity through modeling the pixel intensities in the segmented tumor regions. Each image contains 200×201 gray scale pixels with intensity ranging from 0 to 255. The tumor region in each image is extracted and the pixel intensities in the tumor region is used to calculate the dendrogram tree by agglomerative clustering. Figure 3 shows an example of a T2‐weighted MRI slice, together with the segmented tumor region and a histogram plot of the pixel intensities in that region. As stated in Section 3.2, tumor heterogeneity is related to the distribution spikiness of the pixel intensities in the tumor image, here roughly depicted by the histogram. Our modeling approach provides an easy way to represent tumor heterogeneity by the exponential rate parameter of the bMBP, and associate it with the candidate covariates. The detailed procedure is summarized in the following steps:

1. For each patient, we perform agglomerative clustering (with Euclidean distance, complete linkage) to the pixel intensities in the tumor image. The clustering algorithm starts by treating each pixel as a singleton cluster, and then successively merge pairs of clusters with similar intensity values until all clusters have been merged into one big cluster. Similar agglomerative clustering has been adopted previously in the testing of tree‐structured data.^20^ The clustering results are summarized in a dendrogram—a binary tree with varying branch lengths.
2. By treating the dendrogram tree as a sample path of the bMBP, we calculate the interarrival times in the sample path and treat them as the response variable in (5). These interarrival times are sufficient statistics for the underlying exponential rate parameter of the bMBP.
3. The interarrival times and candidate covariates corresponding to each patient are then used in the proposed GLM. Inference can be performed to identify covariates associated with tumor heterogeneity and estimate their effects, as described in Section 2.2.

Using backward elimination, 10 out of the total 19 covariates were selected, including two demographic variables (age and gender), six trait‐related variables (Karnofsky score, months of disease‐specific survival, FLAIR volume, classical, mesenchymal, and proneural), and two genetic variables (*DDIT3* and *PIK3CA*). Table 4, columns 2 and 3 list the estimated covariate effects (with 95% confidence intervals) for the selected variables. The last column of Table 4 lists the estimates by Lasso for GLM. We see that, Lasso for GLM identified a different set of eight covariates. Among the total 19 covariates, six have been selected by both backward elimination and Lasso for GLM, including two demographic variables (age and gender), three trait‐related variables (Karnofsky score, FLAIR volume, and mesenchymal), and one genetic variables (*PIK3CA*).

**TABLE 4:** Estimated covariate effects (with 95% CIs) for selected variables in the brain‐tumor image data

Parameter	Estimate	95% CI	Estimate by Lasso
Intercept	$4.14\times 10^{-2}$	$\left[3.07\times 10^{-2},\;5.36\times 10^{-2}\right]$	$2.59\times 10^{-2}$
Age	$7.51\times 10^{-5}$	$\left[5.58\times 10^{-5},\;9.73\times 10^{-5}\right]$	$1.08\times 10^{-4}$
Karnofsky score	$-1.84\times 10^{-5}$	$\left[-2.38\times 10^{-5},\;-1.36\times 10^{-5}\right]$	$-1.20\times 10^{-5}$
Gender	$1.12\times 10^{-2}$	$\left[8.30\times 10^{-3},\;1.45\times 10^{-2}\right]$	$5.39\times 10^{-3}$
Months of disease‐specific survival	$3.14\times 10^{-5}$	$\left[2.33\times 10^{-5},\;4.07\times 10^{-5}\right]$	‐
FLAIR volume	$-8.63\times 10^{-8}$	$\left[-1.12\times 10^{-7},\;-6.41\times 10^{-8}\right]$	$-3.95\times 10^{-8}$
Classical	$3.37\times 10^{-3}$	$\left[2.50\times 10^{-3},\;4.37\times 10^{-3}\right]$	‐
Mesenchymal	$-1.16\times 10^{-2}$	$\left[-1.51\times 10^{-2},\;-8.62\times 10^{-3}\right]$	$-1.63\times 10^{-3}$
Proneural	$-7.39\times 10^{-3}$	$\left[-9.57\times 10^{-3},\;-5.48\times 10^{-3}\right]$	‐
*DDIT3*	$1.04\times 10^{-2}$	$\left[7.75\times 10^{-3},\;1.35\times 10^{-2}\right]$	‐
*PIK3CA*	$1.96\times 10^{-3}$	$\left[1.46\times 10^{-3},\;2.54\times 10^{-3}\right]$	$5.62\times 10^{-4}$
*EGFRmut*	‐	‐	$4.61\times 10^{-3}$
*MDM4*	‐	‐	$4.84\times 10^{-4}$

The interpretability of the estimated parameters in the GLM is important as it provides biological meaningful results. From Table 2, we see that the two demographic variables and one genetic variable selected by both methods have positive effects on the exponential rate parameter of bMBP, meaning that patients with larger values in these variables have on average shorter interarrival times or branch lengths in the dendrogram tree, which suggest lower tumor heterogeneity. On the other hand, the three trait‐related variables (Karnofsky score, FLAIR volume, mesenchymal) have negative effects, with larger values indicating higher tumor heterogeneity. In addition, the selected gene, *PIK3CA*, has been previously found to be related to GBM. In literature, *PIK3CA* was widely known to have high frequency mutations to promote GBM pathogenesis.^18^, ^21^, ^22^

## 5 CONCLUSIONS AND DISCUSSION

In this article, we propose to test association between binary trees and a set of covariates. The association testing is done via modeling binary trees by a bMBP and linking its rate parameter to covariates through a GLM framework. We note that, the recent work by Behr et al^6^ also looked into the problem of testing for dependence on tree structures. However, their association model treated the tree structure as the predictor and considered its association with only one response variable, whereas our model treats the tree structure as the response and considers multiple predictors. Simulation studies showed that the statistical inference based on our proposed model, including stepwise regression and Lasso for GLM, achieved satisfactory results. Furthermore, by simulations with semi‐synthetic, model‐free data, we confirmed the applicability of the proposed model on real brain‐tumor image data. Such a modeling and inference approach was finally applied to the MRI data from GBM patients to identify associated covariates and estimate their effects on brain‐tumor heterogeneity. Despite the relatively small sample size of the real data used in this study, six out of a total of 19 covariates were found to be associated with brain‐tumor heterogeneity, including the previously identified gene *PIK3CA*. Overall, the proposed approach is effective in testing association between binary‐tree structured data and covariates. Findings from this study may be used to further investigate the etiology of brain tumor, and gain improvements in assessment and treatment of this disease.

It is noteworthy that the two inference procedures used in our simulation study and real application, backward elimination and Lasso for GLM, both have strengths and limitations. Backward elimination is easy to implement, but its variable selection result may be path dependent especially in the existence of collinearity. Lasso for GLM is more computationally efficient than stepwise regression, but its variable selection result highly depends on the choice of the tuning parameter. In general, both methods can be used for low dimensional variable selection problems such as the one raised from our real application. But Lasso for GLM has the superiority for the large *p* small *n* paradigm and performs better in cross validation as its regularization prevents overfitting.

When modeling binary tree structured data in real applications by the bMBP, we recommend to always check the goodness‐of‐fit of the model by scrutinizing the empirical CDF of the scaled interarrival times. We note that, when the empirical CDF suffers from lack of fit to exponential, it is possible to extend the modeling approach to the non‐Markovian case. Under a more general setting when the branch lengths of the binary tree do not necessarily follow exponential, we may model the binary tree by an age‐dependent branching process (ie, Bellman‐Harris process^23^). The distribution of such an age‐dependent branching process at a given time may be obtained (eg, numerically) by solving a nonlinear integral equation (integrating with respect to the life time distribution).^24^ Using the relation between $S_{n}$ and N(t) (see Section 2.1), the CDF of $S_{n}$ can be obtained as a function of the life time distribution. Thus, we may similarly build a GLM to associate the waiting times $S_{n}$ with covariates through a set of unknown parameters—the life time distribution.

The implication of our application on brain‐tumor image data is to identify clinical or genetic factors that affect brain‐tumor heterogeneity. The binary tree obtained from clustering pixel intensities in the tumor image indicates distinct phenotypic (gray level) patterns of the tumor cells thus provides a good representation of tumor heterogeneity. Other data summaries of the tumor image, such as brightness and contrast, also carry information about tumor heterogeneity. However, the clustering dendrogram tree reveals more latent structures of the brain‐tumor image. For example, pixels with the same ancestor (parent nodes) may reflect tumor cells that are potentially originated from the same etiological source or at the similar developmental stage. We believe that such latent structures carry important information and deserve careful considerations in statistical modeling.

When using the clustering dendrogram tree to represent tumor heterogeneity, the spatial location of the pixels in the image has not been taken into account. With this representation, pixels that are distant can still have the same ancestor as long as their pixel intensities are close. Therefore, such binary‐tree structured data are suitable to indicate the overall heterogeneity of data points that are exchangeable. It is an interesting problem for future study to model the heterogeneity of data points while taking into account spatial correlations.

## Figures and Tables

**FIGURE B1:**
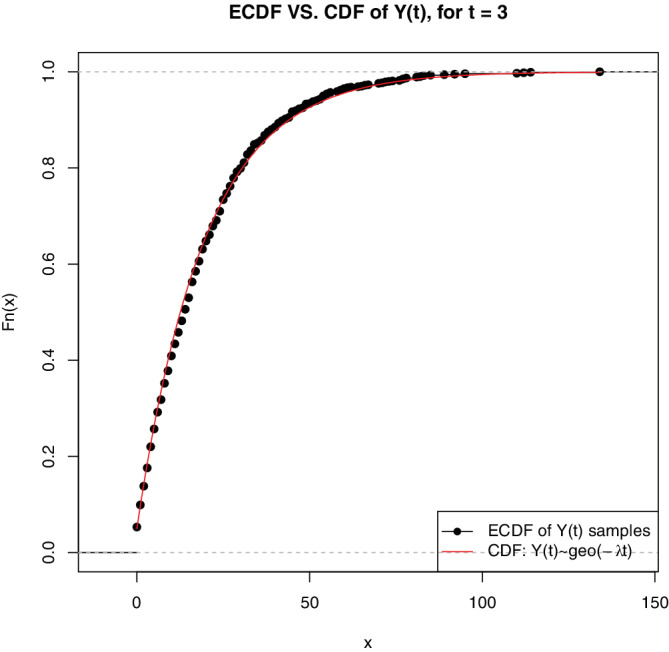
Distribution of Y(t) when t=3, based on 1000 simulated sample paths

**FIGURE B2:**
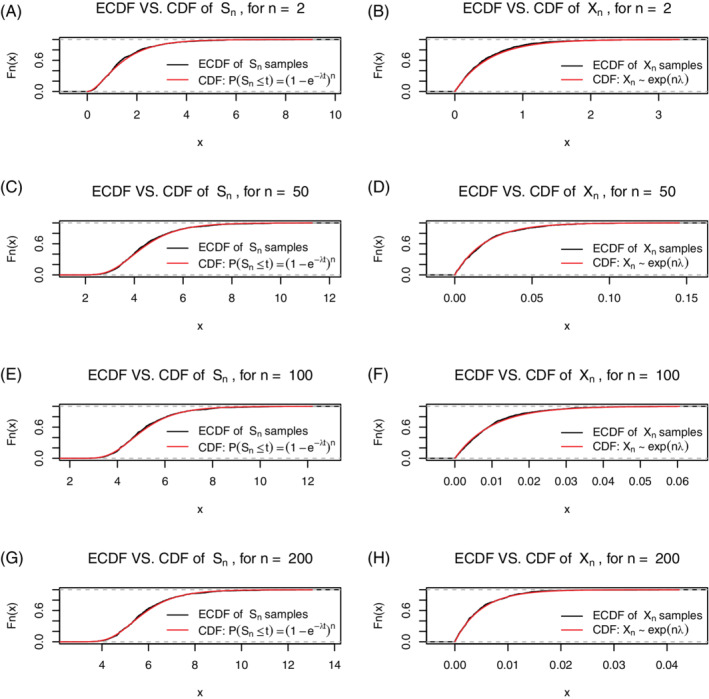
Distributions of $S_{n}$ and $X_{n}$ under different number of birth events n=2,50,100,200, based on 1000 simulated sample paths

**FIGURE B3:**
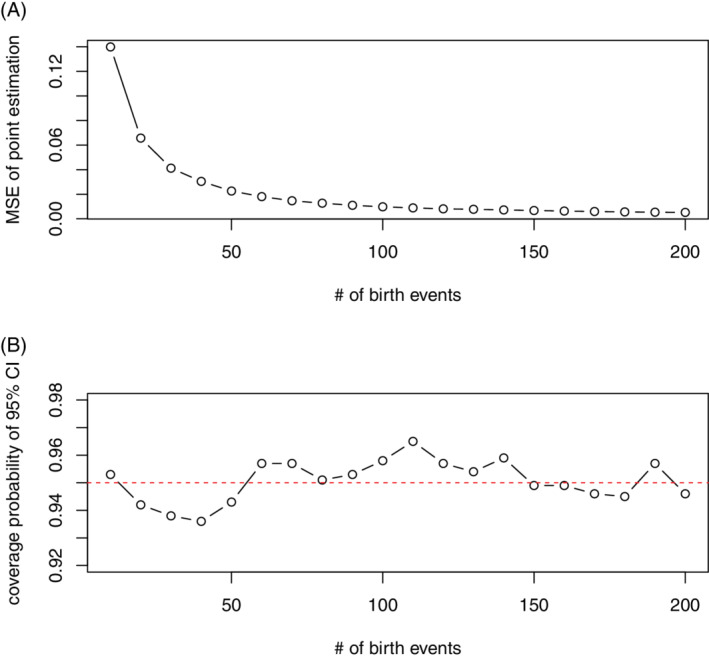
MSE (Panel A) of $\hat{\lambda }$ and coverage probability (Panel B) of 95% CI under different number of birth events, based on 1000 simulated sample paths. The dashed red line in Panel B indicates exact 95% coverage probability

**FIGURE B4:**
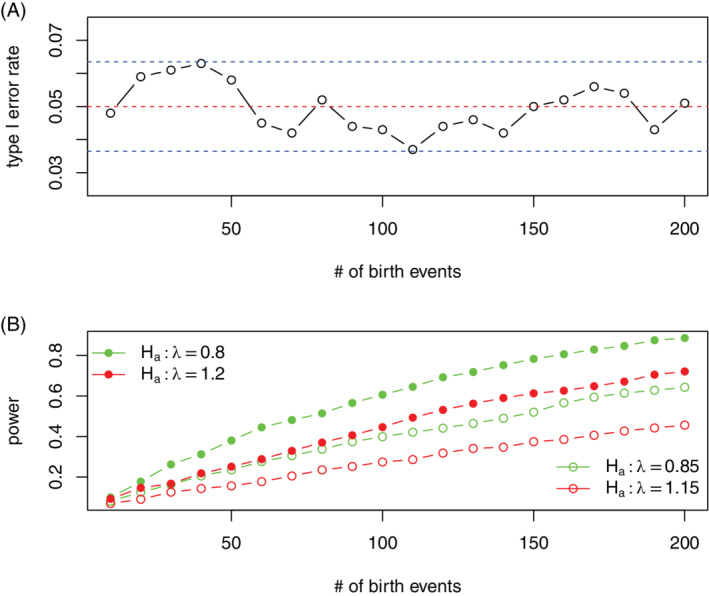
Type I error rate (Panel A) and empirical power (Panel B) of single‐sample LRT under different number of birth events, based on 1000 simulated sample paths. In Panel A, the dashed red line indicates nominal level α=0.05, and the two dashed blue lines provide the upper and lower bounds of the type I error by a *z*‐test

**FIGURE B5:**
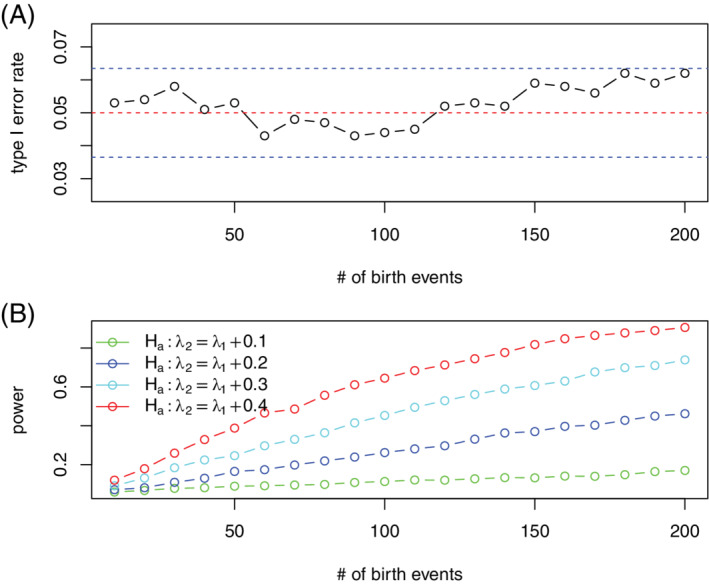
Type I error rate (Panel A) and empirical power (Panel B) of two‐sample LRT under different number of birth events, based on 1000 simulated sample paths. In Panel A, the dashed red line indicates nominal level α=0.05, and the two dashed blue lines provide the upper and lower bounds of the type I error by a *z*‐test

## APPENDIX A INFERENCE ON THE PARAMETER OF BINARY FISSION MBP

### A.1 Parameter estimation

Easy to see that both the maximum likelihood estimator (MLE) and the method of moments (MOM) estimator of parameter λ take the form

(A1)
$$\begin{array}{ll} \hat{\lambda }=\frac{n}{\sum_{i=1}^{n}iX_{i}}. & \end{array}$$

Consistency of this estimator can be shown by checking the moments of $\hat{\lambda }$.

The 100(1−α)% CI of this point estimator is

(A2)
$$\begin{array}{ll} \left[\frac{\hat{\lambda }}{q_{1-\frac{\alpha }{2}}},\frac{\hat{\lambda }}{q_{\frac{\alpha }{2}}}\right], & \end{array}$$

where $q_{\frac{\alpha }{2}}$ represents the α/2 quantile of distribution IG(n,n).

### A.2 Hypothesis testing

Hypothesis testing on λ can be done by likelihood ratio test (LRT). In a single‐sample test, the observed interarrival times from one binary fission MBP are used to test $H_{0}:\lambda =\lambda_{0}$ vs $H_{a}:\lambda \neq \lambda_{0}$; whereas in a two‐sample test, two sets of observed interarrival times, each from a binary fission MBP, are used to test $H_{0}:\lambda_{1}=\lambda_{2}$ vs $H_{a}:\lambda_{1}\neq \lambda_{2}$. For both tests, the LRT statistic

(A3)
$$\begin{array}{ll} \Lambda_{LRT}=-2(\ell_{0}-\ell_{a}), & \end{array}$$

has an asymptotic null distribution of $\chi_{1}^{2}$, where $\ell_{0}$ and $\ell_{a}$ are the log‐likelihoods under $H_{0}$ and $H_{a}$, respectively.

## APPENDIX B A SIMULATION STUDY TO EVALUATE THE INFERENCE RESULTS FOR THE EXPONENTIAL RATE PARAMETER OF bMBP

In this simulation study, we first generate 1000 sample paths from a binary fission MBP with λ=1. These samples are then used to check the distribution of Y(t) for t=3, and the distribution of $S_{n}$ and $X_{n}$ for n=2, 50, 100, 200. The empirical CDFs are shown in Figures B1 (for Y(t)) and B2 (for $S_{n}$ and $X_{n}$), which indeed show a perfect match to the corresponding theoretical CDFs.

Next, we use the 1000 simulated sample paths to evaluate the point and interval estimations of the unknown parameter λ in terms of MSE and coverage probability, respectively. The calculation of MSE and coverage probability is based on different number of birth events n=10, 20,…,200. These results are shown in Figure B3, Panels A (for MSE) and B (for coverage probability). We see that, as *n* increases, the MSE drops down quickly, and the coverage probability of the 95% confidence interval (CI) stabilizes around its theoretical value 0.95, as expected.

Finally, we demonstrate the performance of LRT through simulations, including both single‐sample and two‐sample tests. The type I error rates and empirical powers for the two tests are shown in Figures B4 and B5, respectively. In these simulations, we set different λ values under $H_{a}$. Specifically, the single‐sample test is for $H_{0}:\lambda =1$ vs $H_{a}:\lambda =0.8,\;0.85,\;1.15,\;1.2$, and the two‐sample test is for $H_{0}:\lambda_{1}=\lambda_{2}=1$ vs $H_{a}:\lambda_{1}=1\;\text{and}\;\lambda_{2}=1.1,\;1.2,\;1.3,\;1.4$. We see that, for both tests, the type I error rates are always close to the nominal level despite the setting of *n* (Panel A in Figures B4 and B5), whereas the empirical powers increase with *n*. Also, the empirical powers are influenced by the magnitude of the effect: a larger effect size leads to higher power (shown by different line colors and markers in Panel B in Figures B4 and B5).

## APPENDIX C A SIMULATION STUDY TO EVALUATE STEPWISE REGRESSION BY THE LRT CRITERION

Stepwise regression involves an iterative process of selecting between neighboring models. Under the GLM framework (5) and (4), we consider selecting between two models: the full model in which $\beta_{k}\neq 0$, for all k∈{1,…,q} vs the partial model in which $\beta_{l}=0$ for some l∈{1,…,q} and $\beta_{k}\neq 0$ for k≠l. The model selection can be done by a LRT. Denote the log‐likelihood of the partial model by $\ell_{p}$ (or equivalently, $\ell_{0}$ under the null hypothesis), and the log‐likelihood of the full model by $\ell_{f}$ (or $\ell_{a}$ under the alternative), the LRT statistic

$$\begin{array}{ll} \Lambda_{\text{LRT}}=-2(\ell_{p}-\ell_{f}) & \end{array}$$

has an asymptotic null distribution of $\chi_{\text{df}}^{2}$ where df is the number of zero valued β coefficients in the partial model. Next, by independence between the exponentially distributed interarrival times, the log‐likelihood $\ell_{f}$ can be obtained

$$\begin{array}{llll} \ell_{f} & =\overset{m}{\underset{i=1}{\sum }}\overset{n}{\underset{j=1}{\sum }}\left[\ln\left(j\lambda_{i}^{\left(f\right)}\right)-j\lambda_{i}^{\left(f\right)}x_{ij}\right]\; & & \\ & =m\ln n!+n\overset{m}{\underset{i=1}{\sum }}\ln\lambda_{i}^{\left(f\right)}-\overset{m}{\underset{i=1}{\sum }}\left(\lambda_{i}^{\left(f\right)}\overset{n}{\underset{j=1}{\sum }}jx_{ij}\right),\; & & \end{array}$$

where $\lambda_{i}^{\left(f\right)}=\beta_{0}^{\left(f\right)}+\sum_{k=1}^{q}\beta_{k}^{\left(f\right)}Z_{ik}$. Similarly, $\ell_{p}$ can be obtained as a function of $\beta_{k}^{\left(p\right)},0\leq k\neq l\leq q$.

In this simulation study, we consider binary‐tree structured data for 1000 patients. Two covariates, age and gender, are included in the proposed model described in (3) and (4). The age variable, denoted by $Z_{1}$, is sampled uniformly from [18, 80], and the gender variable, denoted by $Z_{2}$, is generated from Bernoulli(0.5). We set $\beta_{0}=0.015,\beta_{1}=0.0003,\beta_{2}=0.22$. Based on the prescribed exponential rate parameter, sample paths are generated from a bMBP for the 1000 patients. This model is denoted as Model I: the “true” model.

In order to check the performance of variable selection by LRT, we consider two alternative models, namely, Model II and Model III. Model II is a “redundant” model, in which an additional non‐associated variable $Z_{3}$ is included besides the two associated covariates $Z_{1}$ and $Z_{2}$. This newly added variable is sampled from folded normal distribution with mean 0 and standard deviation 0.1. Model III includes only one associated covariate, either $Z_{1}$ or $Z_{2}$, and therefore can be treated as a “reduced” model. We then select between Models I and II (“true” vs “redundant”), and between Models I and III (“true” vs “reduced”). Such a simulation and model selection procedure is repeated 100 000 times, and the accuracy of variable selection is calculated by counting how many times the two associated covariates $Z_{1}$ and $Z_{2}$ are correctly selected. Throughout the simulations, we set the upper bound *n* of the number of splitting events as n=100, and use a nominal level α=0.05. We found that, among the 100 000 repeats, 742 selected Model II (“redundant”) over Model I (“true”), none selected Model III (“reduced,” including $Z_{1}$ or $Z_{2}$ only) over Model I (“true”). This suggests that the variable selection by LRT is indeed effective (from a hypothesis testing perspective, the type I error rate, 0.00742, is well controlled and the empirical power is 1). For all 100 000 simulations with correctly selected variables in Model I, we further calculate the MLE of the coefficients $\beta_{0},\beta_{1}$, and $\beta_{2}$. The corresponding mean squared errors (MSE) are $2.19\times 10^{-6}$, $9.13\times 10^{-10}$, and $6.32\times 10^{-6}$, respectively. The coverage probabilities of the 95% CI are 0.9546, 0.95, and 1, respectively.
